# Prepartal Stress, Prepartal and Postpartal Hair Glucocorticoid Concentrations, and Symptoms of Postpartum Depression 3 Days and 12 Weeks After Delivery

**DOI:** 10.1016/j.bpsgos.2025.100454

**Published:** 2025-01-29

**Authors:** Dena Sadeghi-Bahmani, Serge Brand, Gunther Meinlschmidt, Marion Tegethoff, Jennifer Kurath, Nicole Bürki, Irene Hösli, Thorsten Mikoteit

**Affiliations:** a Department of Psychology, Stanford University, Stanford, California; b Department of Epidemiology and Population Health, Stanford University, Stanford, California; c Psychiatric Clinics of the University of Basel, Centre for Affective, Stress and Sleep Disorders, Basel, Switzerland; d Department of Sport and Health Science, Faculty of Medicine, University of Basel, Sport Science Section, Basel, Switzerland; e Kermanshah University of Medical Sciences, Department of Psychiatry, Substance Abuse Prevention Research Center, Health Institute, Kermanshah, Iran; f Kermanshah University of Medical Sciences, Department of Psychiatry, Sleep Disorders Research Center, Kermanshah, Iran; g Tehran University of Medical Sciences, School of Medicine, Tehran, Iran; h Center for Disaster Psychiatry and Disaster Psychology, Center of Competence of Disaster Medicine of the Swiss Armed Forces, Basel, Switzerland; i Clinical Psychology and Psychotherapy, Methods and Approaches, Department of Psychology, Trier University, Trier, Germany; j Department of Digital and Blended Psychosomatics and Psychotherapy, Psychosomatic Medicine, University Hospital Basel and University of Basel, Basel, Switzerland; k Institute of Psychology, RWTH Aachen University, Aachen, Germany; l Department for Gynecology and Obstetrics, Hospital of Baselland, Liestal, Switzerland; m Clinic for Gynecology and Obstetrics, Hospital of the University of Basel, Basel, Switzerland; n Psychiatric Services of Solothurn and University of Basel, Solothurn, Switzerland

**Keywords:** Hair cortisol, Hair cortisone, Hypothalamic-pituitary-adrenal (HPA) axis, Life events, Postpartum depression (PPD), Pregnancy

## Abstract

**Background:**

Postpartum depression (PPD) is a serious mental health problem that affects about 17% of mothers. The aims of the current study were to observe the associations between prenatal stress, self- and expert-rated PPD, and prepartal and postpartal hair cortisol and cortisone concentrations as proxies for altered hypothalamic-pituitary-adrenal axis activity (HPA-AA).

**Methods:**

A total of 129 mothers (mean age 33.1 years) completed the Edinburgh Postnatal Depression Scale 3 days (baseline) and 12 weeks (study end) postpartum. At the end of the study, participants reported on prepartum stressful life events, experts rated participants’ symptoms of depression, and participants provided 6 cm of hair strands for analysis of hair glucosteroid levels 12 weeks before and 12 weeks after delivery.

**Results:**

Prepartal stress was associated with higher scores for self- and expert-rated PPD and with lower hair cortisone concentrations as a proxy for less adaptive HPA-AA. Higher prepartal and postpartal hair cortisol/cortisone ratios (i.e., higher cortisol/lower cortisone concentrations) were associated with higher PPD symptom scores.

**Conclusions:**

Women with prepartal stress were at increased risk of experiencing PPD 12 weeks after delivery. Altered hair steroid levels (lower cortisone concentrations) as a proxy for altered HPA-AA further substantiated this association. Results suggest that 1) both prepartal stress and the suppression of HPA-AA appear to be involved in the development of PPD; 2) hair steroid analysis can be used to predict PPD; and 3) women with prepartal stressful life events may benefit from timely support and relief to decrease their risk of developing PPD.

## Plain Language Summary

Postpartum depression is a severe psychiatric problem that affects about 17% of mothers. We showed among 129 mothers (mean age 33.1 years) that prenatal stress was associated with a higher risk of experiencing symptoms of self- and expert-rated postpartum depression 12 weeks after delivery. Lower hair cortisone concentrations and higher hair cortisol/hair cortisone ratios accompanied this association. Thus, psychological ratings and physiological processes were associated. Given that standardized programs to cope with (prenatal) stress are available, pregnant women with high stress may benefit from such interventions to decrease their risk of postpartum depression.

Some days after delivery, 40% to 80% of mothers experience symptoms of depression (the "baby blues") (1), and about 17% to 20% (2) experience postpartum depression (PPD) during the first 6 months after delivery (1,3, 4, 5, 6, 7). Women with PPD report symptoms of depressed mood, emotional tension, agitation, irritability, intrusiveness and hostility, loss of activity and energy, social withdrawal, feelings of worthlessness and guilt, poorer cognitive performance, sleep disturbances, and suicidal behavior (1,8). Stressful life events appear to be the main drivers of PPD (1). PPD is regarded as a major depressive disorder, with the specifier of onset 4 weeks after delivery (8).

### Psychosocial and Stress-Related Factors and Postpartum Depression

Identified predictors of PPD include unexpected pregnancies, lack of social and partner support (9, 10, 11, 12, 13), violence and abuse, immigration status, negative birth experiences, multiple births (12), preexisting or history of psychopathology (anxiety, depression), and drug use (1).

### Stress, Hypothalamic-Pituitary-Adrenal Axis Activity, and PPD

The cortisol hypothesis/hypothalamic-pituitary-adrenal axis activity (HPA-AA) is a psychophysiological model for depression according to which the occurrence of stress unfavorably impacts psychophysiological processes (14,15) in general and HPA-AA specifically (12,16, 17, 18). The key outcome of HPA-AA is the cortisol concentration.

#### Hypercortisolemia, Hypocortisolemia, and PPD

Four reviews and meta-analyses have shown mixed results for the PPD–HPA-AA link (1,12,18,19). Among those, Mlili *et al.* (18) identified 2 studies with a higher hair cortisol–higher PPD link, 1 study with a lower hair cortisol–higher PPD link, and 4 studies with no hair cortisol–PPD link. Possible confounders were sleep disturbances, concomitant symptoms of depression and anxiety, medications, substance use, diet, the lack of or insufficient social support, and methodological issues such as the cortisol sampling method (blood, saliva, hair), sampling time variations (16,17), and the definition and measurement of stress, which is understood to be a highly individualized cognitive-emotional process (20), which may further explain the PPD–HPA-AA disconnect (17). Lang *et al.* (21) were unable to identify an association between hair cortisol concentrations, stress, and dimensions of psychopathology among 240 mothers in the first 3 months after birth. Marceau *et al.* (22) suspected inconsistencies in the description of data gathering, laboratory analyses, and data elaboration, and King *et al.* (23) did not observe associations between hair cortisol concentrations and depression among 85 participants during pregnancy or at 6 months postpartum.

Hypocortisolemia was observed in saliva from pregnant women with major depressive disorder during the second and third trimester (24) and in women with PPD (25,26), and lower hair cortisol concentrations pre- and postpartum have been observed in women with PPD (27). Hypercortisolemia was observed among pregnant women with longer-lasting symptoms of depression (16), suggesting that hypocortisolemia appears to reflect chronically dysregulated and blunted HPA-AA, while hypercortisolemia reflects currently adaptive HPA-AA. Furthermore, while higher hair cortisol concentrations were observed among pregnant women who scored high on psychiatric symptoms during the third trimester (state condition), poorer symptomatic functioning (trait condition) was associated with lower hair cortisol concentrations (28). In the current study, we assessed hair cortisol and cortisone concentrations as retrospective indicators of pre- and postpartal HPA-AA.

### Glucocorticoid Measurement in Hair

Assessing cortisol and cortisone concentrations in hair has become standard (29, 30, 31). While sex, age, hair washing frequency, hair treatment, and contraceptive use may all modify hair cortisol levels (30), hair cortisol concentrations are robust across a broad range of confounding influences, such as sex, hair color, age, and hair treatment (32,33). Caparros-Gonzalez *et al.* (34) assessed 44 postpartum women at either low or high risk for PPD. Hair strands were taken 16 days after delivery, which reflected the hair cortisol concentrations of the entire pregnancy, and hair cortisol concentrations increased continuously in women without symptoms of PPD from the first to the third trimester (34), while hair cortisol concentrations were consistently higher in women with symptoms of PPD. Consistent with this, higher hair cortisol concentrations were associated with more symptoms of subjective pregnancy-specific stress and more severe symptoms of depression and anxiety. By contrast, Braig *et al.* (33) were unable to find any association between hair cortisol concentrations and self-reported symptoms of chronic stress, anxiety, or depression in a sample of 768 participants. To explain such null findings, Braig *et al.* (33) argued that the completion of self-rating questionnaires about 1.5 days after delivery might have biased participants’ recall from long-term memory. They (33) also reviewed previous studies and concluded that symptoms of anxiety and depression might be associated with both hypo- and hyperactivity of HPA-AA, as measured by hair cortisol concentrations.

Orta *et al.* (35) assessed hair cortisol concentrations in 97 pregnant women, separately for all 3 trimesters, and observed that higher scores on measures of general anxiety and perceived stress were associated with lower hair cortisol concentrations, confirming previous findings (30,36). Lower hair cortisol concentrations were also associated with chronic stress (37, 38, 39, 40), generalized anxiety disorder (41,42), and posttraumatic stress disorder (43, 44, 45, 46).

### The Role of Cortisone as the Bioinactive Glucocorticoid of the Bioactive Cortisol

During pregnancy, increased cortisol concentrations as a proxy for maternal physiological stress may increase the risk of negatively impacting the fetus’ development, including intrauterine growth restriction, reduced birth weight, and postpartal developmental behavior and health (47, 48, 49). Thus, glucocorticoids are regulating signals for the fetus’ intrauterine development (50). To protect the fetus from detrimental effects of maternal cortisol, the enzyme 11β-HSD2 metabolizes the bioactive maternal and potentially detrimental cortisol into the bioinactive cortisone. While 11β-HSD2 is prevalent ubiquitously, the enzyme is especially important in the placenta, and its activity is responsible for this oxidation process as early as in the middle of the first trimester (51,52) (also see the Supplement). In the current study, we considered both prepartal and postpartal cortisol/cortisone ratios as proxies for adaptive HPA-AA, and we asked whether and to what extent prepartal and postpartal cortisol/cortisone ratios were associated with symptoms of PPD.

Four hypotheses were formulated. First, following others (1,12,17), we predicted that women who reported more prepartal stress during pregnancy would be at higher risk of having higher scores on a measure of PPD 3 days and 12 weeks after delivery. Second, following the hypocortisolemia hypothesis (16,24, 25, 26), we predicted that women with higher scores on a measure of prepartal stress would show lower cortisol concentrations as a proxy for less flexible and blunted HPA-AA. Last, following others (25,53,54), we expected that a higher pre- and postpartal cortisol/cortisone ratio would be associated with higher PPD symptom scores, both self-rated (hypothesis 3) and expert-rated (hypothesis 4).

## Methods and Materials

### Procedure

Mothers who had recently given birth at the Department of Gynecology and Obstetrics of the University Hospital Basel (Basel, Switzerland) or at the Department of Gynecology and Obstetrics at the General Hospital of Liestal (Liestal, Switzerland) were approached on postpartum day 3 and asked to take part in the current consecutive study of the associations between stressful life events during pregnancy, PPD, and hair steroid levels. Eligible participants were fully informed about the aims of the study and the confidential data handling. Thereafter, participants signed written informed consent forms. At baseline (3 days after delivery) and at the end of the study (12 weeks after delivery), participants completed the Edinburgh Postnatal Depression Scale (EPDS) and the Inventory of Life Changing Events (ILE) to assess critical life events during pregnancy (now labeled prepartal stress). Experts rated participants’ depression severity (Hamilton Depression Rating Scale [HDRS]) at the end of the study. Next, again at the end of the study and as an indicator of hair steroid levels of the last 12 weeks before and the first 12 weeks after delivery, participants gave 6 cm of hair strands. The local ethics committee (Ethikkommission Nordwest- und Zentralschweiz; study registration: EKBB 82/12 Basel, Switzerland) approved the study, which was performed according to the Declaration of Helsinki (55).

### Participants

A total of 146 mothers participated in the study. Inclusion criteria were age ≥18 years, term delivery (≥37 weeks of gestation), willing and able to comply with the study conditions, and provided written informed consent. Exclusion criteria were acute severe medical or mental disorder (e.g., acute addiction of alcohol or illegal drugs, acute psychosis, acute suicidal ideation) as assessed by an experienced psychiatrist and based on a thorough clinical interview (56), severe complications during pregnancy or delivery (e.g., rescue cesarean section), and withdrawal from the study. A planned cesarean section was not an exclusion criterion.

### Measures

#### Sociodemographic Information

Participants reported on their age (years), civil status (single, married), educational level (compulsory school education, higher education), and current job situation (unemployed, employed).

#### Obstetric Characteristics

The following information was extracted from medical records: number of deliveries (primiparae or multiparae), gestational week at delivery, and planned cesarean section (yes or no).

#### Self-Report of Depression on the EPDS

Three days and 12 weeks after delivery, participants completed the EPDS (57). It consists of 10 items, and higher total scores reflect higher self-rated symptoms of PPD. The maximum score is 30, and a score of ≥10 is considered as PPD (58) (Cronbach’s α = 0.91).

#### Expert Ratings of Depression on the HDRS

Experts rated participants’ depression severity with the HDRS (59). This gold-standard measure consists of 17 items (60,61), although given that 3 items assess sleep disturbances and that a newborn’s sleep-wake pattern modulates the mother’s sleep-wake pattern, these 3 items were deleted. In doing so, we avoided biasing the HDRS total score simply because of the mother’s altered sleep schedules. Answers are given on a 3- to 5-point Likert scale ranging from 0 (never/not at all) to 3 to 4 (always; very high), with higher total scores reflecting more marked depressive symptoms. Categories are as follows: 0 to 7 = no depressive symptom/remission, 8 to 17 = mild depression, 18 to 24 = moderate depression, and ≥25 = severe depression (Cronbach’s α = 0.87).

#### Severe Life Changing Events During Pregnancy: ILE

To assess subjectively experienced stress exposure during pregnancy, participants completed the ILE (62), which was adapted for pregnancy (63) (see the Supplement). One continuous dimension and 5 dichotomous variables were derived from this questionnaire.

### Hair Strand Sampling

To sample hair strands, we followed the recommendations of Kirschbaum *et al.* (64) [see the Supplement and (27)].

#### Assessment of Hair Steroids

As has been described elsewhere (27,65), hair steroids were assessed by the biochemical Laboratory of the University of Dresden (Germany) using liquid chromatography–tandem mass spectrometry (see the Supplement).

### Statistical Analyses

With a series of Pearson’s correlations, we estimated whether PPD symptom scores were associated, both self- and expert-rated and after 3 days and 12 weeks after delivery.

Similarly, we ran repeated-measures analyses of variance (ANOVAs) with prepartal stress (yes or no) and time points (3 days and 12 weeks after delivery) as factors and symptoms of PPD as dependent variables. For expert-rated symptoms of PPD 12 weeks after delivery, we performed *t* tests.

Using a series of Pearson’s correlations, we tested whether higher scores for prepartal stress were associated with altered hair steroid concentrations (cortisol, cortisone, cortisol/cortisone ratio). Likewise, we ran repeated-measures ANOVAs with prepartal stress (yes or no) and time points (12 weeks before and 12 weeks after delivery) as factors and hair glucosteroid concentrations as dependent variables.

A series of repeated-measures ANOVAs was performed to examine whether self- and expert-rated (yes or no) diagnoses of PPD and time points (12 weeks before and 12 weeks after delivery) were systematically linked with the patterns of hair steroid concentrations.

The level of significance was set at α < .05. All computations were performed with SPSS version 29.0 (IBM Corp.) for Apple Mac.

## Results

### Sample Characteristics

While a total of 146 mothers participated in the study, sufficient hair steroid data were available from 133 women (mean age 33.0 years; SD 4.9). Of those, 4 had no data on self- and expert-assessed depression; the final sample size was 129 participants. Mean length of gestation was 39.4 weeks (SD 1.4 weeks); 59 participants (44.1%) were primiparae; and 52 participants (40.3%) had a planned cesarean section. All participants except 1 (99.1%) were cohabitating with their partners, and 59 participants (45.7%) had a university degree.

### Preliminary Calculations

Maternal age and length of gestation were statistically unrelated to symptoms of depression (self-ratings 3 days and 12 weeks after delivery, expert-ratings 12 weeks after delivery), prepartal stress (continuous variable), and hair steroid concentrations (cortisol, cortisone) (all *r*s < 0.10, *p*s > .40).

Primiparae versus multiparae state and mode of delivery (vaginal delivery vs. cesarean section) were not systematically related to symptoms of depression (self-ratings 3 days and 12 weeks after delivery, expert-ratings 12 weeks after delivery), prepartal stress (continuous variable), or hair steroid concentrations (cortisol, cortisone) (all *r*s < 0.10, *p*s >.40). Therefore, maternal age, length of gestation, parity, and mode of delivery were not introduced as confounders.

### Prepartal Stress and Symptoms of Depression After Delivery

#### Correlations Between Perceived Prepartal Stress and Self- and Expert-Rated Symptoms of Depression 3 Days and 12 Weeks After Delivery

Table 1 shows Pearson’s correlation coefficients between prepartal stress and self- and expert-rated symptoms of PPD.

**Table 1:** Correlation Coefficients for Prepartal Stress and Symptoms of Depression After Delivery (*n* = 89)

		1	2	3	4	5	Mean (SD)
1	Perceived prepartal stress, continuous variable	–	0.34∗∗	0.45∗∗∗	0.46∗∗∗	0.51∗∗∗	3.18 (3.12)
2	PPD 3 days after delivery^a^	–	–	0.56∗∗∗	0.51∗∗∗	0.54∗∗∗	5.55 (4.08)
3	PPD 12 weeks after delivery^a^	–	–	–	0.68∗∗∗	0.74∗∗∗	5.07 (4.26)
4	Expert ratings of symptoms of depression 12 weeks after delivery, 17 items^b^	–	–	–	–	0.98∗∗∗	3.20 (5.41)
5	Expert ratings of symptoms of depression 12 weeks after delivery, 14 items^b^	–	–	–	–	–	2.18 (4.42)

PPD, postpartum depression.

∗∗*p* < .01, ∗∗∗*p* < .001.

a Assessed with the Edinburgh Postnatal Depression Scale.

b Assessed with the Hamilton Depression Rating Scale, 17 or 14 items, respectively.

Higher perceived prepartal stress was associated with higher scores for self-rated symptoms of PPD both 3 days and 12 weeks after delivery, and with higher expert-rated scores for PPD 12 weeks after delivery. Higher PPD symptom scores 3 days after delivery were associated with higher scores for self-rated PPD 12 weeks after delivery and with higher scores for expert-rated depression 12 weeks after delivery. Higher scores for self-rated PPD 12 weeks after delivery were associated with higher scores for expert-rated depression 12 weeks after delivery. Overall, higher scores for prepartal stress and symptoms of self- and expert-rated depression were associated at 3 days and 12 weeks after delivery.

#### Prepartal Stress (Dichotomous Variable) and Symptoms of Depression 3 Days and 12 Weeks After Delivery

Five repeated-measures ANOVAs were performed with perceived prepartal stress (first trimester = ILE 1, second trimester = ILE 2, third trimester = ILE 3, second and third trimester = ILE 2 + 3, total pregnancy = ILE 1 + 2 + 3) as factors (yes or no) and self-rated symptoms of depression 3 days and 12 weeks after delivery as dependent variables.

Descriptive and inferential statistical indices for self- and expert-rated symptoms of depression are reported in Table 2. Irrespective of the time lapse, women who reported perceived prepartal stress also had higher depression symptom scores both 3 days and 12 weeks after delivery.

**Table 2:** Descriptive and Inferential Statistical Indices for Self-Rated Postpartal Symptoms of Depression, Separately by Time and Group

ILE	Perceived Stress During Pregnancy				Factors		
	Yes		No				
	Symptoms of Depression		Symptoms of Depression				
	3 Days After Delivery	12 Weeks After Delivery	3 Days After Delivery	12 Weeks After Delivery	Time, *F*, η_p_^2^	Group, *F*, η_p_^2^	Time × Group Interaction, *F*, η_p_^2^
ILE 1	33, 6.48 (5.01)	6.24 (4.78)	33, 4.88 (3.16)	3.79 (2.85)	1.88, 0.028 S	5.35∗, 0.80 M	0.76, 0.028 S
ILE 2	33, 6.48 (4.64)	6.30 (4.80)	33, 4.45 (3.09)	3.85 (2.71)	0.77, 0.012 S	6.87∗∗, 0.10 M	0.22, 0.003 S
ILE 3	32, 6.63 (4.56)	6.16 (4.79)	32, 4.16 (2.91)	3.84 (2.76)	0.86, 0.014 S	7.56∗∗, 0.11 M	0.03, 0.001 S
ILE 2 + 3	37, 6.51 (3.04)	6.00 (4.63)	28, 4.25 (3.04)	3.61 (2.39)	1.99, 0.031 S	7.00∗∗, 0.10 M	0.03, <0.001 S
ILE 1 + 2 + 3	43, 5.84 (4.51)	5.81 (4.38)	25, 4.44 (2.97)	3.53 (2.45)	1.23, 0.019 S	4.30∗, 0.061 S	1.16, 0.017 S

Values are presented as *n*, mean (SD) or mean (SD).

∗*p* < .05, ∗∗*p* < .01.

The Inventory of Life Changing Events (ILE), as a measure of perceived stress, was analyzed for the first trimester (ILE 1), second trimester (ILE 2), third trimester (ILE 3), second and third trimesters (ILE 2 + 3), and entire pregnancy (ILE 1 + 2 + 3). S indicates small effect size; M indicates medium effect size.

Using a series of *t* tests, we examined whether participants with and without prepartal stress (ILE; dichotomous variable) differed with regard to symptoms of depression, as rated by experts 12 weeks after delivery. The descriptive and inferential statistical indices are reported in Table 3.

**Table 3:** Descriptive and Inferential Statistical Indices of Expert-Rated Postpartal Symptoms of Depression 12 Weeks After Delivery

ILE	Perceived Stress During Pregnancy				*t* Tests	
	Yes		No			
	Expert Depression Rating		Expert Depression Rating			
	17 Items	14 Items	17 Items	14 Items	17 Items	14 Items
ILE 1	33, 5.70 (7.80)	4.30 (6.55)	32, 1.91 (2.30)	0.91 (1.30)	*t*_63_ = 2.64∗∗, *d* = 0.66 M	*t*_63_ = 2.88∗∗, *d* = 0.75 M
ILE 2	34, 4.74 (5.85)	3.71 (4.69)	32, 1.75 (2.37)	0.75 (1.27)	*t*_64_ = 2.68∗∗, *d* = 0.66 M	*t*_64_ = 3.44∗∗∗, *d* = 0.85 L
ILE 3	34, 6.29 (8.43)	5.12 (6.99)	30, 1.60 (2.37)	0.67 (1.24)	*t*_62_ = 2.95∗∗, *d* = 0.74 M	*t*_62_ = 3.44∗∗∗, *d* = 0.86 L
ILE 2 + 3	40, 5.75 (7.89)	4.60 (6.59)	26, 1.73 (2.51)	0.69 (1.29)	*t*_64_ = 2.51∗∗, *d* = 0.63 M	*t*_64_ = 2.98∗∗, *d* = 0.75 M
ILE 1 + 2 + 3	44, 5.36 (7.63)	4.18 (6.41)	23, 1.83 (2.60)	0.78 (1.35)	*t*_65_ = 2.15∗∗, *d* = 0.55 M	*t*_65_ = 2.51∗∗, *d* = 0.64 M

Values are presented as *n*, mean (SD) or mean (SD).

∗∗*p* < .01, ∗∗∗*p* < .001.

The Inventory of Life Changing Events (ILE), as a measure of perceived stress, was analyzed for the first trimester (ILE 1), second trimester (ILE 2), third trimester (ILE 3), second and third trimesters (ILE 2 + 3), and entire pregnancy (ILE 1 + 2 + 3). M indicates medium effect size; L indicates large effect size.

Irrespective of the number of items (17 vs. 14), women who reported perceived prepartal stress were rated by experts as experiencing more severe symptoms of depression 12 weeks after delivery.

### Hair Steroids Over Time and Between and Within Specific Samples

Table 4 shows the prenatal and postnatal mean cortisol and cortisone concentrations for the total sample.

**Table 4:** Mean Prenatal and Postnatal Cortisol and Cortisone Concentrations for the Total Sample (*n* = 129)

	Time Points	
	Prenatal	Postnatal
Cortisol, pg/mg	0.89 (0.58)	0.92 (0.38)
Cortisone, pg/mg	1.14 (0.45)	1.52 (0.46)

Values are presented as mean (SD).

#### Prepartal Stress (Dichotomous Variable) and Pre- and Postpartal Hair Cortisol Concentrations

Using a series of repeated-measures ANOVAs, we tested whether participants with and without prepartal stress (ILE; dichotomous variable) differed in their cortisol concentrations.

Irrespective of the time lapse of perceived prepartal stress, pre- and postpartal hair cortisol concentrations did not statistically vary over time and within or between participants with and without prepartal stress (all *F*_1,__127_ < 3.00, *p*s > .50, η_p_^2^ < 0.02).

#### Self-Rated PPD 12 Weeks After Delivery (Yes or No) and Prepartal and Postpartal Hair Cortisol Concentrations

Using a repeated-measures ANOVA, we tested whether participants with and without self-rated PPD 12 weeks after delivery showed different hair cortisol concentrations prepartally and postpartally. No descriptive or statistically significant associations of mean hair cortisol concentrations prepartally or postpartally with or without self-rated PPD were observed (all *F*_1,127_ < 1.50, *p* > .38).

#### Expert-Rated PPD 12 Weeks After Delivery (Yes or No) and Prepartal and Postpartal Hair Cortisol Concentrations

Using a repeated-measures ANOVA, we tested whether participants with and without expert-rated PPD 12 weeks after delivery showed different hair cortisol concentrations prepartally and postpartally. No descriptive or statistically significant mean hair cortisol concentrations prepartally or postpartally with or without expert-rated PPD were observed (all *F*_1,127_ < 1.50, *p* > .39).

Overall, hair cortisol concentrations did not statistically or systematically vary from the pre- to the postpartum period, in relation to prepartal stress, or as a function of self- or expert-rated PPD.

#### Prepartal Stress (Dichotomous Variable) and Pre- and Postpartal Hair Cortisone Concentrations

Using a series of repeated-measures ANOVAs, we tested the hypothesis that participants with and without prepartal stress (ILE; dichotomous variable) would differ with regard to hair cortisone concentrations. Irrespective of the time lapse of perceived prepartal stress, hair cortisone concentrations increased from the prepartal to the postpartal stage. Compared with participants who reported no prepartal stress during the first trimester, participants who reported prepartal stress during the first trimester had statistically lower hair cortisone concentrations (*F*_1,127_ = 3.42, *p* < .05, η_p_^2^ = 0.096; medium effect size).

#### Self-Rated PPD 12 Weeks After Delivery (Yes or No) and Prepartal and Postpartal Hair Cortisone Concentrations

Hair cortisone concentrations increased over time (*F*_1,127_ = 18.94, *p* < .01, η_p_^2^ = 0.249; large effect size), and compared with participants with no self-rated PPD, participants with self-rated PPD 12 weeks after delivery had lower hair cortisone concentrations (*F*_1,127_ = 4.48, *p* < .01, η_p_^2^ = 0.073; medium effect size).

#### Expert-Rated PPD 12 Weeks After Delivery (Yes or No) and Prepartal and Postpartal Hair Cortisone Concentrations

Irrespective of expert-rated PPD status (yes or no), hair cortisone concentrations increased over time (*F*_1,127_ = 10.13, *p* < .01, η_p_^2^ = 0.156; large effect size). Overall, hair cortisone concentrations increased from the pre- to the postpartal stage. Participants with prepartal stress and self-reported PPD had lower hair cortisone concentrations than their counterparts.

#### Prepartal Stress (Dichotomous Variable) and Pre- and Postpartal Hair Cortisol/Cortisone Ratio Concentrations

Using a series of repeated-measures ANOVAs, we tested whether participants with and without prepartal stress (ILE; dichotomous variable) differed with regard to hair cortisol/cortisone ratio concentrations. Except for the total score for prepartal stress over the entire pregnancy, hair cortisol/cortisone ratios decreased statistically significantly over time (*F*_1,127_ = 9.91–18.43, *p*s < .01, η_p_^2^ = 0.261–0.236; always large effect sizes), while the factor prepartal stress (yes or no) was not significantly associated with cortisol/cortisone ratios. Closer inspection of the descriptive data showed that the decrease in the cortisol/cortisone ratio was mainly driven by an increase in cortisone concentrations.

#### Self-Rated PPD 12 Weeks After Delivery (Yes or No) and Prepartal and Postpartal Hair Cortisol/Cortisone Ratios

Using repeated-measures ANOVA, we tested whether participants with and without self-reported PPD differed with regard to hair cortisol/cortisone ratio concentrations. The hair cortisol/cortisone ratio decreased statistically significantly over time (*F*_1,127_ = 8.33, *p* < .01, η_p_^2^ = .122; medium effect size). Compared with participants with no self-reported PPD, participants with PPD had a higher cortisol/cortisone ratio (*F*_1,127_ = 7.10, *p* < .01, η_p_^2^ = 0.106; medium effect size). Closer inspection of the descriptive data showed that the increase in the cortisol/cortisone ratio in participants with self-rated depression was driven by the decrease in cortisone.

#### Expert-Rated PPD 12 Weeks After Delivery (Yes or No) and Prepartal and Postpartal Hair Cortisol/Cortisone Ratios

Using repeated-measures ANOVA, we tested the hypothesis that participants with and without expert-rated PPD would differ regarding hair cortisol/cortisone ratio concentrations.

The hair cortisol/cortisone ratio decreased statistically significantly over time (*F*_1,127_ = 15.68, *p* < .001, η_p_^2^ = 0.236; large effect size) in the total sample. Compared with participants with no expert-rated PPD, participants with PPD had a higher cortisol/cortisone ratio (*F*_1,127_ = 7.46, *p* < .01, η_p_^2^ = 0.114; medium effect size). Closer inspection of the descriptive data indicated that the increase in the cortisol/cortisone ratio in participants with depression was driven by a decrease in cortisone.

Overall, hair cortisol/cortisone ratios decreased over time in the total sample, although hair cortisol/cortisone ratios were higher among participants with self-reported and expert-rated symptoms of PPD. Inspection of the descriptive data indicated that the increase in the cortisol/cortisone ratio was driven by a decrease in cortisone.

## Discussion

The aims of the current study were to estimate whether and to what extent prenatal stress was associated with symptoms of PPD 3 days and 12 weeks after delivery and whether such patterns could be further substantiated with hair steroids (cortisol, cortisone, cortisol/cortisone ratio concentrations) as proxies for pre- and postpartal HPA-AA. The key findings were as follows. First, higher scores for perceived prenatal/prepartal stress were associated with higher self- and expert-rated PPD symptom scores 3 days and 12 weeks after delivery. Second, higher prepartal stress was associated with altered HPA-AA both prenatally and postnatal/postpartally. Lastly, higher pre- and postnatal cortisol/cortisone concentration ratios were associated with higher scores for prenatal stress and both self-rated and expert-rated PPD. Importantly, higher cortisol/cortisone ratios were mainly driven by decreased cortisone concentrations.

Four hypotheses were formulated, and each of these is considered now in turn. With the first hypothesis, we assumed that women who reported more prepartal stress were at higher risk of reporting higher PPD symptom scores 3 days and 12 weeks after delivery, and the data confirmed this (see Table 1). Accordingly, we confirmed what has been observed elsewhere (1,12,17). The current results expand upon the current literature in the following 4 ways. First, both correlational and inferential computations (prepartal stress yes or no as factor) indicated that higher prepartal stress scores predicted higher PPD scores. Second, higher self-rated PPD scores were observed as early as 3 days after delivery, and such scores remained stably high 12 weeks after delivery. Third, self-rated prepartal stress also predicted higher expert-rated PPD scores. Fourth, data highlighted the chronological and continuous setup of a woman’s self-reported and expert-rated symptoms of depression after delivery as a function of what she is experiencing as psychosocially burdensome throughout her pregnancy.

According to the second hypothesis, we assumed that women with higher prenatal stress scores would show lower cortisol concentrations as a proxy for altered HPA-AA, although our data could not confirm this expectation for hair cortisol, but only for hair cortisone. First, women with high prenatal stress also reported lowered prenatal and postnatal hair cortisone concentrations. Second, women with no prenatal stress also had higher prenatal and postnatal hair cortisone concentrations, and these women (third) also showed an increase in hair cortisone concentrations from the prenatal to the postnatal stage. With this, we could confirm the so-called hypocortisolemia hypothesis (16,24, 25, 26), and we replicated what had been observed before among 48 women with PPD compared with women with no PPD (27).

With the third hypothesis, we expected that higher pre- and postpartal cortisol/cortisone ratios would be associated with higher scores for self-rated PPD, and our data confirmed this: Both higher prepartal and postpartal cortisol/cortisone ratios were associated with higher PPD scores. With these findings, we confirmed previous results (66).

With the fourth hypothesis, we expected that higher pre- and postpartal cortisol/cortisone ratios would be associated with higher scores for expert-rated PPD, and our data confirmed this. Therefore, we confirmed again what has been described elsewhere (66).

The specific patterns of cortisol/cortisone ratios deserve closer attention. First, results showed that a decreased cortisol/cortisone ratio was mainly driven by an increase in cortisone concentrations instead of by changes in cortisol concentrations. Such an increase in cortisone concentrations should be considered to be a favorable neuroendocrine process of the maternal organism. Increased cortisol concentrations as a proxy for maternal physiological stress may increase the risk of a negative impact on the fetus’ development, including intrauterine growth restriction, reduced birth weight, and postpartal developmental behavior and health (47, 48, 49). Thus, glucocorticoids are regulating signals for the fetus’ intrauterine development (50). However, as mentioned above, the enzyme 11β-HSD2 is responsible for the oxidation process to metabolize the bioactive maternal cortisol concentrations into the bioinactive cortisone as early as in the middle of the first trimester (51,52). Thus, our data indicated that pregnant and postpartum women with prepartal stress, above all those with symptoms of depression, showed lower cortisone concentrations and, as a result, increased cortisol/cortisone ratios. This pattern may have resulted from decreased 11β-HSD2 activity in chronically stressed participants and participants with depression, although the quality of the data did not allow a direct evidence. However, the pattern of results is consistent with the current literature, which indicates that prepartal stress can downregulate 11β-HSD2 activity, potentially resulting in an increased cortisol/cortisone ratio by lower cortisone concentrations (16,67,68).

### Limitations and Future Directions

First, critical life events during pregnancy, labeled as prepartal stress, were retrospectively assessed in the current study. Given this, we acknowledge the risk of bias due to current mood state, as it was well described in the seminal work on mood-congruent memory bias (69, 70, 71). Second, sleep and anxiety were not assessed even though perinatal states and PPD are associated with impaired sleep (72, 73, 74) and anxiety (75,76). Third, social support appeared to have a stress-buffering effect via the downregulation of stress responses, including dampened sympathetic activity and HPA-AA (75), and the perceived social support of the husband was the strongest predictor of low symptoms of depression (77, 78, 79); future studies should consider these factors. Fourth, infants’ sleep, feeding, and behavioral characteristics were not assessed, although mothers of newborns with infantile colic reported impaired sleep and higher depression symptom scores (80,81). Next, the inconsistent results in the HPA-AA-PPD link research may also be partially attributed to discrepancies in the time of assessment [see (29,30,38,82)]. Last, given that symptoms of stress were retrospectively assessed during pregnancy but not symptoms of depression, we cannot fully rule out the possibility that participants with higher stress scores prenatally and higher depression scores postnatally did not already experience symptoms of depression during the pregnancy.

### Conclusions

Women who reported higher scores for prepartal stress were at increased risk of experiencing PPD. Such a pattern of association was further substantiated by specific patterns of hair steroid concentrations (especially lower hair cortisone and higher hair cortisol/hair cortisone), highlighting the psychophysiological link of pregnancy, the postpartum stage, and the endocrine stress system in the development of PPD. Given that standardized intervention programs to cope with stress are available, pregnant women with higher stress levels may benefit from such psychotherapeutic programs.

## Supplementary Material

Supplemental Text

Key Resources Table
