# Supplementary material for: Prepartal Stress, Prepartal and Postpartal Hair Glucocorticoid Concentrations, and Symptoms of Postpartum Depression 3 Days and 12 Weeks After Delivery
Source: Biol Psychiatry Glob Open Sci. 2025 Jan 29;5(3):100454. doi: 10.1016/j.bpsgos.2025.100454 (PMC11925523; doi:10.1016/j.bpsgos.2025.100454)
Supplement: Supplemental Text [file mmc1.pdf]

SUPPLEMENTARY INFORMATION

Prepartal Stress, Prepartal and Postpartal Hair Glucocorticoid Concentrations, and Symptoms of Postpartum Depression 3 Days and 12 Weeks After Delivery

Sadeghi-Bahmani *et al.*

Supplementary material:

Introduction: Cortisol/cortisone ratio

11β-HSD2 is produced and secreted in the syncytial layer of the placental vili and in fetal membranes (1) and does not bind to glucocorticoid receptors (GR) and mineraloglucocorticoid receptors (MR) (2-4). In this line, cortisone is not protein-bound, i.e. cortisone levels are independent from the cortisol binding globulin (CBG), and accordingly, measurements of cortisone in plasma or saliva reflect the total concentration of cortisone. Observations from animal and human studies (5) showed that the 11β-HSD2 insufficiency was related with pregnancy adversity such as pre-eclampsia, intra-uterine growth restriction and preterm birth. Further, cortisone increases during the second and third trimester (6).

As a result, and plausibly, a higher cortisol/cortisone-ratio means: A higher amount of cortisol is available, compared to cortisone, which is understood as an unfavorable neuroendocrinological process, most probably due to the 11β-HSD2 insufficiency.

2.3.5. Severe life changing events during pregnancy; Inventory of Life Changing Events (ILE); henceforth labelled as prepartal stress

To assess subjectively experienced stress exposure during pregnancy, participants completed the Inventory of Life Changing Events (ILE) (7)), adapted for pregnancy (8). The pregnancy-adapted version of the ILE explores the occurrence of 32 pre-defined (and one not-predefined 'other') negative life events during pregnancy, such as death of spouse or a close family member, personal injury or illness, change in financial state, unemployment, and similar. Answers are given on four-points rating scales ranging from 1 (= not at all burdensome) to 4 (= very burdensome), with higher sum scores reflecting a more stressfully perceived pregnancy. Besides, the following dichotomous were: "one or more subjectively stressful life events during the first trimester" labelled ILE 1; "one or more subjectively stressful life events during the second trimester" labelled ILE 2, and so on (see (8).Thus, one continuous dimension and five dichotomous variables were derived from this questionnaire.

2.4. Hair strands sampling

To sample hair strands, we followed the recommendations of Kirschbaum et al. (9) (see supplementary materials: (9) and (10)]. The steps were as follows: 1. trained staff members divided a participant's hair at the back of the head using a hair grip. 2. two to three hair strands are separated close to the participants' scalp in the posterior vertex region; hair strands should be at least 3 mm in diameter; as a rule of thumb, 3mm equal to half of the diameter of a pencil. 3. hair strands are combed. 4. combed hair strands are bundled with a prepared packthread loop. 5. 6cm of strands are cut as close as possible to the scalp. 6. strands are placed in prepared aluminum foil. 7. the scalp near end with the packthread loop is clearly marked with a water-proof marker. 8. the aluminum foil is folded and both ends are sealed. 9. all aluminum foils are consecutively numbered and labeled.

The 6cm of hair closest to the scalp were tested for steroid concentrations and provided a value reflecting hair steroid concentrations for the last 2 x 12 weeks, that is to say, 12 weeks before and 12 weeks after delivery (assuming an average hair growth rate of 1cm/month). All participants had long hair and could provide sufficient material for analysis. Note that 3cm hair segments close to the scalp correspond to a more recent period in time (postpartum), while 3cm segments distant from the scalp correspond to a more distant period (antepartum). Consequently, hair strands were cut in 2 x 3cm, corresponding to the two time periods. The two 3cm hair strands were separately analyzed.

As described elsewhere (10, 11), hair steroids were assessed by the biochemical Laboratory of the University of Dresden (Germany) using mass spectrometry (LCMS/MS) [see supplementary materials]. Samples were washed in 2.5 mL isopropanol for 3 minutes, and steroid hormones were extracted from 7.5 mg of whole, non-pulverized hair using 1.8 mL methanol in the presence of 50 µL cortisol-d4, cortisone-d7, testosterone-d5, DHEA-d4, and progesterone-d9 as internal standards for 18 hours at room temperature. Samples were spun in a bench top centrifuge (Mikro 22R; Hettich GmbH and Co. KG, Tuttlingen, Germany) at 15,200 g relative centrifugal force for 2 minutes, and 1 mL of the clear supernatant was transferred into a new 2 mL tube. The alcohol was evaporated at 50°C under a constant stream of nitrogen and reconstituted with 225 µL double-distilled water, 50 µL of which were injected into a Shimadzu HPLC-tandem mass spectrometry system (Shimadzu, Canby, Oregon) coupled to an AB Sciex API 5000 Turbo-ion-spray triple quadrupole tandem mass spectrometer (AB Sciex, Foster City, California) with purification by online solid-phase extraction. The lower limits of quantification (LOQ) of this analysis were below 0.1 pg/mg for cortisol and cortisone. The inter- and intra-assay coefficients of variance were below 7% for cortisol, and below 8% for cortisone.

1. Zhu P, Wang W, Zuo R, Sun K. Mechanisms for establishment of the placental glucocorticoid barrier, a guard for life. Cell Mol Life Sci. 2019;76(1):13-26. Epub 20180917. doi: 10.1007/s00018-018-2918-5. PubMed PMID: 30225585; PubMed Central PMCID: PMC11105584.
2. Seckl JR. Glucocorticoids, feto-placental 11 beta-hydroxysteroid dehydrogenase type 2, and the early life origins of adult disease. Steroids. 1997;62(1):89-94. doi: 10.1016/s0039-128x(96)00165-1. PubMed PMID: 9029721.
3. Seckl JR. Prenatal glucocorticoids and long-term programming. Eur J Endocrinol. 2004;151 Suppl 3:U49-62. doi: 10.1530/eje.0.151u049. PubMed PMID: 15554887.
4. Seckl JR, Meaney MJ. Glucocorticoid "programming" and PTSD risk. Ann N Y Acad Sci. 2006;1071:351-78. doi: 10.1196/annals.1364.027. PubMed PMID: 16891583.
5. Konstantakou P, Mastorakos G, Vrachnis N, Tomlinson JW, Valsamakis G. Dysregulation of 11beta-hydroxysteroid dehydrogenases: implications during pregnancy and beyond. J Matern Fetal Neonatal Med. 2017;30(3):284-93. Epub 20160419. doi: 10.3109/14767058.2016.1171308. PubMed PMID: 27018008.
6. Wilson M, Thayer Z. Maternal salivary cortisone to cortisol ratio in late pregnancy: An improved method for predicting offspring birth weight. Psychoneuroendocrinology. 2017;78:10-3. Epub 20170112. doi: 10.1016/j.psyneuen.2016.12.018. PubMed PMID: 28131073.
7. Siegrist J, Geyer S. Inventar zur Erfassung lebensverändernder Ereignisse. In: Brähler E, Schumacher B, ;, Strauss B, editors. Diagnostische Verfahren in der Psychotherapie. Göttingen: Hogrefe; 2002. p. 211-3.

8. Tegethoff M, Raul JS, Jamey C, Khelil MB, Ludes B, Meinlschmidt G. Dehydroepiandrosterone in nails of infants: a potential biomarker of intrauterine responses to maternal stress. Biol Psychol. 2011;87(3):414-20. Epub 20110607. doi: 10.1016/j.biopsycho.2011.05.007. PubMed PMID: 21645584.
9. Kirschbaum C, Tietze A, Skoluda N, Dettenborn L. Hair as a retrospective calendar of cortisol production-Increased cortisol incorporation into hair in the third trimester of pregnancy. Psychoneuroendocrinology. 2009;34(1):32-7. Epub 2008/10/25. doi: 10.1016/j.psyneuen.2008.08.024. PubMed PMID: 18947933.
10. Jahangard L, Mikoteit T, Bahiraei S, Zamanibonab M, Haghighi M, Sadeghi Bahmani D, et al. Prenatal and Postnatal Hair Steroid Levels Predict Post-Partum Depression 12 Weeks after Delivery. J Clin Med. 2019;8(9). Epub 2019/08/28. doi: 10.3390/jcm8091290. PubMed PMID: 31450789.
11. Gao W, Stalder T, Foley P, Rauh M, Deng H, Kirschbaum C. Quantitative analysis of steroid hormones in human hair using a column-switching LC-APCI-MS/MS assay. Journal of chromatography B, Analytical technologies in the biomedical and life sciences. 2013;928:1-8. Epub 2013/04/16. doi: 10.1016/j.jchromb.2013.03.008. PubMed PMID: 23584040.
